# Sensitive Electrochemical Immunosensor for Detection of Nuclear Matrix Protein-22 based on NH_2_-SAPO-34 Supported Pd/Co Nanoparticles

**DOI:** 10.1038/srep24551

**Published:** 2016-04-18

**Authors:** Dan Wu, Yaoguang Wang, Yong Zhang, Hongmin Ma, Tao Yan, Bin Du, Qin Wei

**Affiliations:** 1 Key Laboratory of Chemical Sensing & Analysis in Universities of Shandong, School of Chemistry and Chemical Engineering, University of Jinan, Jinan 250022, P.R. China; 2 School of Resources and Environment, University of Jinan, Jinan 250022, P.R. China

## Abstract

A novel sandwich-type electrochemical immunosensor using the new amino group functionalized silicoaluminophosphates molecular sieves (NH_2_-SAPO-34) supported Pd/Co nanoparticles (NH_2_-SAPO-34-Pd/Co NPs) as labels for the detection of bladder cancer biomarker nuclear matrix protein-22 (NMP-22) was developed in this work. The reduced graphene oxide-NH (rGO-NH) with good conductivity and large surface area was used to immobilize primary antibody (Ab_1_). Due to the excellent catalytic activity toward hydrogen peroxide, NH_2_-SAPO-34-Pd/Co NPs were used as labels and immobilized secondary antibody (Ab_2_) through adsorption capacity of Pd/Co NPs to protein. The immunosensor displayed a wide linear range (0.001–20 ng/mL) and low detection limit (0.33 pg/mL). Good reproducibility and stability have showed satisfying results in the analysis of clinical urine samples. This novel and ultrasensitive immunosensor may have the potential application in the detection of different tumor markers.

Bladder cancer (BC) is one of the most common urinary cancers^1^. For the clinical diagnosis of human bladder cancer, cystoscopy is considered to be the gold standard^2^,^3^. However, the cystoscopy is expensive, invasive^4^,^5^ and it has difficulties in detecting upper urinary tract lesions. Therefore, it is necessary to develop a noninvasive, quick and highly sensitive method for the detection of BC. Nuclear matrix protein-22 (NMP-22) is a nuclear mitotic apparatus protein which is involved in the proper distribution of chromatids to daughter cells during cellular replication^6^,^7^,^8^. NMP-22 is widely used as a tumor marker for bladder tumor, and is involved with DNA recombination and replication, RNA transcription and mitosis^5^,^9^,^10^. Numerous studies show that the level of NMP-22 is usually less than 5 ng/mL, and 80% of terminal bladder cancer people have high levels of NMP-22^11^,^12^. NMP-22 is thought to be an objective, noninvasive, quantitative test with good accuracy in BC diagnosis, especially for low-grade tumors^12^,^13^. NMP-22 has become increasingly significant in the detection of bladder cancer and is being used for the diagnosis and detection of recurrence^13^.

Reduced graphene oxide (rGO), a two-dimensional nanomaterial consisted of sp^2^-hybridized carbon atoms to form a one-atom thick honeycomb lattice, has been considered as a promising candidate for electron-acceptor and electron-transfer material due to its excellent optical and electrical properties, which has been extensively studied in the field of electrochemical immunoassay^14^,^15^,^16^,^17^,^18^. Moreover, the populated chemical moieties on the rGO surface offer the convenience and flexibility for various functionalizations to enhance the sensor performance. More importantly, rGO may also be functionalized through covalent or non-covalent methods in order to further enhance its sensitivity, specificity, loading capacity, biocompatibility, etc. Reduced graphene oxide-NH (rGO-NH) is a novel material which is a combination of rGO and piperazine through covalent bonding. The rGO-NH not only keeps the original property of rGO but also promotes water solubility.

Ordered mesoporous materials are one kind of molecular sieve which have attracted increasing interest owing to the unique properties which can be effectively applied in electrochemical devices^19^, the fields of catalysis and supported catalysts^20^,^21^,^22^, electroanalytical chemistry^23^,^24^,^25^,^26^,^27^ and biosensors^28^,^29^,^30^,^31^. The silicoaluminophosphates molecular sieves (SAPO-34) with high stability, microporosity, large specific surface area and acid sites^32^,^33^,^34^,^35^ can immobilize more Pd/Co nanoparticles and enhance the sensitivity of immunosensor. Pd-based catalysts have been widely used as catalysts for the direct synthesis of hydrogen peroxide (H_2_O_2_) from oxygen and hydrogen elemental^36^,^37^, and the decomposition of H_2_O_2_ is also catalyzed by Pd-based catalysts^38^,^39^. However, Pd/Co nanoparticles have not been studied for designing electrochemical immunosensor. In this work, the novel amino group functionalized silicoaluminophosphates molecular sieves (NH_2_-SAPO-34) supported Pd/Co nanoparticles (NH_2_-SAPO-34-Pd/Co NPs) were first used as labels of the secondary antibody (Ab_2_).

In this work, a sandwich-type electrochemical immunosensor for the detection of NMP-22 was prepared by using NH_2_-SAPO-34-Pd/Co NPs as labels and rGO-NH as sensing platform for the signal amplification. The large surface area of rGO-NH could increase the loading of Ab_1_ and the good conductivity of rGO-NH could promote the electron transfer. The high catalysis of NH_2_-SAPO-34-Pd/Co NPs toward the reduction of H_2_O_2_ could improve the sensitivity of the immunosensor. Therefore, this simple, economic and sensitive immunosensor could be widely used in the clinical analysis.

## Experimental

### Materials and reagents

NMP-22 antigen and antibody were purchased from Guyan Biotech Co., Ltd. (Shanghai, China). K_3_[Fe(CN)_6_] was purchased from Sinopharm Chemical Reagent Co., Ltd. Glutaraldehydes (GA) and sodium tetrachloropalladate (Na_2_PdCl_4_) were purchased from Sinopharm Chemical Reagent Beijing Co., Ltd. (China). Cobalt nitrate was obtained from Shanghai Chemical Reagent Plant (China). Bovine serum albumin (BSA) and chitosan (CS) were purchased from Sigma-Aldrich. The rGO-NH was obtained from Nano Innova Technologies Co., Ltd. (Spain)^40^. The SAPO-34 was purchased from the catalyst factory of Nankai University (China). Phosphate buffered saline (PBS, 1/15 M Na_2_HPO_4_ and KH_2_PO_4_) was used as electrolyte for all electrochemical measurement. Ultrapure water was used throughout the experiments.

### Apparatus

All electrochemical measurements were performed on a CHI760D electrochemical workstation (Shanghai CH Instruments Co., China). Scanning electron microscope (SEM) and Energy Dispersive X-Ray Spectroscopy (EDS) were recorded by JEOL JSM-6700F microscope (Japan). A conventional three-electrode system was used for all electrochemical measurements: the modified glassy carbon electrode (GCE, 4 mm in diameter) as the working electrode, a saturated calomel electrode (SCE) as the reference electrode, and platinum wire electrode as the counter electrode.

### Preparation of NH_2_-SAPO-34

0.1 g of SAPO-34 powder, 0.1 mL of 3-ammonia propyl triethoxy silane and 10 mL of anhydrous ethanol were added into the three necked flask, the mixture was heated to and kept at 70 °C for 1.5 h. Then the product was cooled down to room temperature and collected by centrifugation (7000 rpm, 5 min). Finally, the product was collected after washing and drying in vacuum at 40 °C.

### Synthesis of NH_2_-SAPO-34-Pd/Co NPs

The NH_2_-SAPO-34-Pd/Co NPs were synthesized by a modified two-step reduction route under the protection of high-purity nitrogen in an ice bath^41^. Firstly, 4 mL of 0.2 mol/L Co(NO_3_)_2_, 2.5 mL of 64 mmol/L sodium citrate, 30 mg of NH_2_-SAPO-34 and 25 mL of ultrapure water were mixed ultrasonically for 20 min. Then, 5 mL of 1.6 mol/L NaBH_4_ solution was added into the above mixture at a rate of 20 mL/h under vigorous stirring to generate the Co NPs. Secondly, 10 mL of 40 mmol/L Na_2_PdCl_4_ solution and 10 mL of 0.16 mol/L NaBH_4_ solution were synchronously added into the solution of Co NPs at a rate of 20 mL/h. Finally, the resulting NH_2_-SAPO-34-Pd/Co NPs was filtrated, washed with ultra-pure water for more than three times and then dried in vacuum at 35 °C.

### Preparation of NH_2_-SAPO-34-Pd/Co-Ab_2_

**Figure 1:**
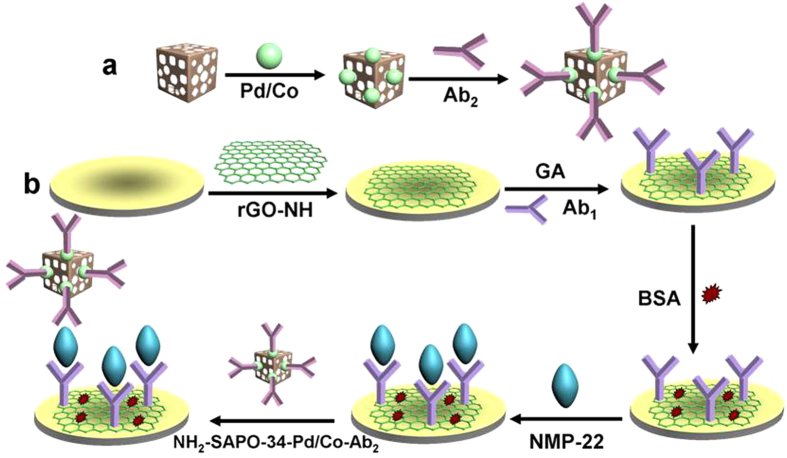
Schematic representation of the preparation of the NH_2_-SAPO-34-Pd/Co-Ab_2_ (**a**) and fabrication process of modified immunosensor (**b**).

The NH_2_-SAPO-34-Pd/Co-Ab_2_ was synthesized by the following steps (Fig. 1(a)). A solution of NH_2_-SAPO-34-Pd/Co NPs (2 mg/mL, 1 mL) was added into Ab_2_ dispersion (10 μg/mL, 1 mL) and stirred for 12 h at 4 °C. After centrifugation, the resulting NH_2_-SAPO-34-Pd/Co-Ab_2_ was dispersed in 1 mL of PBS at pH 7.0 and stored at 4 °C.

### Modification of electrodes

Figure 1(b) showed the fabrication procedure of the immunosensor. A glassy carbon electrode was polished to a mirror-like finish with 1.0, 0.3 and 0.05 μm alumina powder and then thoroughly cleaned. Afterwards, 6.0 μL of rGO-NH solution (1.5 mg/mL) dispersed in chitosan (0.1 wt%) was dropped onto the electrode surface and then dried at room temperature. The utilization of chitosan could make rGO-NH forming a film on the electrode surface and the abundant amino groups in CS could provide active sites for Ab_1_ immobilization. To immobilize the Ab_1_ onto the electrode, 3.0 μL of GA (2.5%, v/v) was dropped onto the electrode surface and incubated until it was half-dry. Then, 6.0 μL of Ab_1_ (10 μg/mL) was dropped onto the electrode surface and then incubated for 1 h. In this procedure, GA was used as cross-linking agent to link amino groups of antibody with amino groups of CS. After drying, the electrode was incubated in 1 wt% BSA solution for another 30 min to eliminate nonspecific binding sites. Subsequently, NMP-22 solution with different concentrations were dropped onto the electrode surface and incubated for 1 h, and the excess antigen was rinsed away with water. Finally, 6.0 μL of the prepared NH_2_-SAPO-34-Pd/Co-Ab_2_ solution was dropped onto the electrode surface and bound to NMP-22 via the specific antibody-antigen interaction. After incubation, the electrode was then rinsed and stored at 4 °C before use. The label NH_2_-SAPO-34-Pd/Co could catalyze the reduction of H_2_O_2_, so that different current response could be generated in accordance with NMP-22 concentration when 10 μL of H_2_O_2_ (5.0 mol/L) was added into 10 mL of PBS under magnetic stirring.

## Results and Discussion

### Characterization of rGO-NH and NH_2_-SAPO-34-Pd/Co NPs

**Figure 2:**
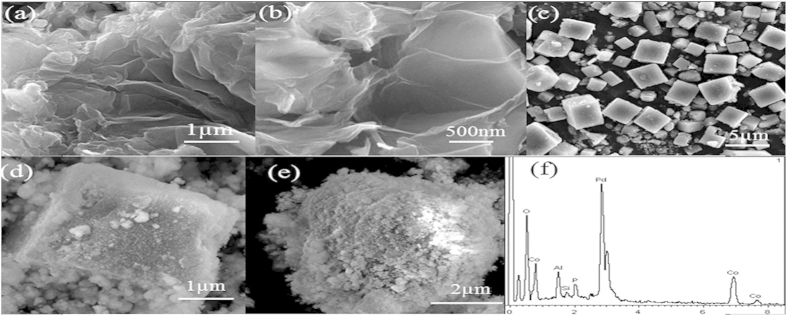
SEM images of rGO-NH (**a,b**) NH_2_-SAPO-34 (**c**) and NH_2_-SAPO-34-Pd/Co NPs (**d,e**); EDS of NH_2_-SAPO-34-Pd/Co NPs (**f**).

The rGO-NH with large surface area was used to increase the amount of captured Ab_1_. The SEM images (Fig. 2a,b) of the rGO-NH showed that the rGO-NH had a wrinkle paper-like structure with irregular size, further indicating the large surface area of rGO-NH. The SEM image of NH_2_**-**SAPO-34 was shown in Fig. 2c, which showed the NH_2_**-**SAPO-34 possessed cubic structure. The NH_2_**-**SAPO-34 had a BET surface area of 536.6 m^2^/g. Due to the large surface area, more Pd/Co nanoparticles could be loaded on the surface of NH_2_**-**SAPO-34 and the SEM images (Fig. 2d,e) of NH_2_-SAPO-34-Pd/Co presented that the NH_2_**-**SAPO-34 was coated successfully. Elemental compositions of NH_2_-SAPO-34-Pd/Co NPs were analyzed by EDS (Fig. 2f). Signature peaks of Si, O, P, Al, Pd and Co were observed, indicating that the Pd/Co nanoparticles were formed successfully on the surface of NH_2_**-**SAPO-34.

### Characterization of NH_2_-SAPO-34-Pd/Co NPs modified electrode

**Figure 3:**
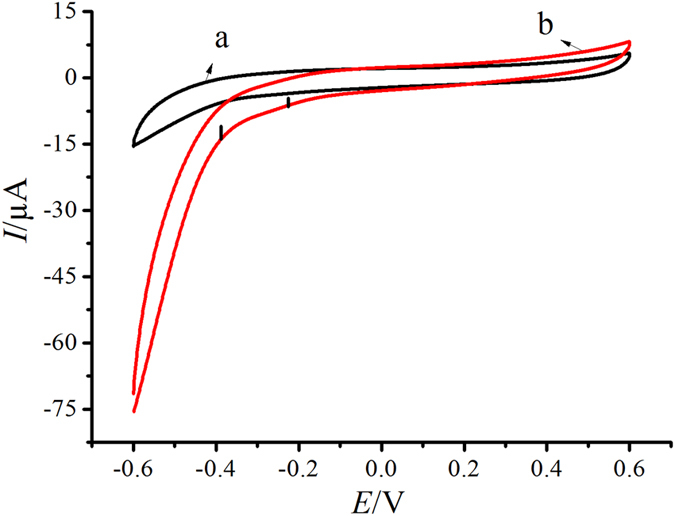
Cyclic voltammograms of electrode modified by NH_2_-SAPO-34-Pd/Co-Ab_2_ (2 mg/mL) in N_2_-saturated PBS without (**a**) and with (**b**) 5.0 mmol/L H_2_O_2_.

The performance of NH_2_-SAPO-34-Pd/Co toward the H_2_O_2_ reduction was investigated (Fig. 3). As shown, the electrode modified by NH_2_-SAPO-34-Pd/Co-Ab_2_ exhibited obvious current change toward H_2_O_2_. However, the electrode did not appear to be electroactive toward water. The NH_2_-SAPO-34-Pd/Co NPs showed the ability to promote the reduction of H_2_O_2_ and resulted in the generation of electrochemical signals.

### Characterization of the immunosensor

**Figure 4:**
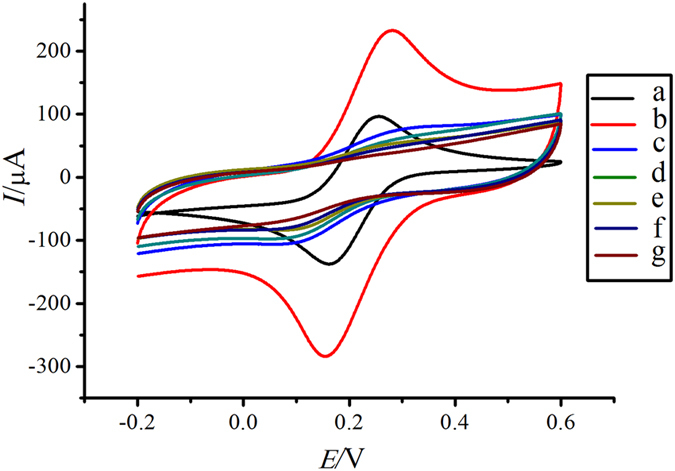
The cyclic voltammetry for each immobilized step in a PBS of pH 7.0 buffer solution containing 5 mmol/L K_3_[Fe(CN)_6_] on the response of the immunosensor to 10 ng/mL NMP-22. The bare GCE (**a**), rGO-NH/GCE (**b**), GA/rGO-NH/GCE (**c**), Ab_1_/GA/rGO-NH/GCE (**d**), BSA/Ab_1_/GA/rGO-NH/GCE (**e**), NMP-22/BSA/Ab_1_/GA/rGO-NH/GCE (**f**) and NH_2_-SAPO-34-Pd/Co-Ab_2_/NMP-22/BSA/Ab_1_/GA/rGO-NH/GCE (**g**).

The stepwise modified process of electrode was characterized by cyclic voltammetry (CV). CV can also characterize the modification process of the immunosensor besides electrochemical impedance spectroscopy, and each immobilization step was shown in Fig. 4. It could be seen that a pair of well-defined redox peak was observed on GCE (curve a), and this quasi-reversible one-electron redox peak was attributed to the transformation between Fe(CN)_6_^4−^ and Fe(CN)_6_^3−^. The redox peak current increased strongly after rGO-NH was dropped onto the electrode surface (curve b), which suggested the rGO-NH had good conductivity and strong ability of electron transfer. The redox peak current decreased significantly after GA was dropped onto the rGO-NH modified electrode (curve c), which could be attributed to the large impedence of GA. The redox peak current decreased gradually when Ab_1_ (curve d), BSA (curve e) and NMP-22 (curve f) as the non-conductive bioactive substances were modified layer by layer on the electrode. The results suggested that the non-conductive bioactive substances were immobilized onto the electrode successfully and blocked electron exchange between the redox probe and the electrode. The redox peak current decreased to the minimum (curve g) when NH_2_-SAPO-34-Pd/Co-Ab_2_ were immobilized, indicated the formation of hydrophobic immunocomplex layer could embarrass electron transfer. As a result, the immunosensor was modified successfully.

### Optimization of experimental conditions

**Figure 5:**
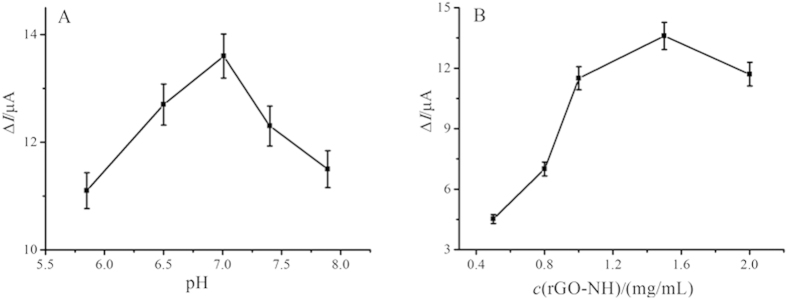
(**a**) Effect of pH (C_rGO-NH_ = 1.5 mg/mL, pH = 5.85, 6.50, 7.00, 7.40 and 7.89) and (**b**) the concentration of rGO-NH (pH = 7.0, C_rGO-NH_ = 0.5, 0.8, 1.0, 1.5 and 2.0 mg/mL) on the response of the immunosensor to 10 ng/mL NMP-22. Error bar = RSD (*n* = 5).

To achieve an optimal electrochemical signal, the influence of the pH value of substrate solution to the immunosensor was investigated at first. Herein, 6.0 μL of rGO-NH solution (1.5 mg/mL) dispersed in chitosan (0.1 wt%) was dropped onto the electrode surface. Then the same amount of Ab_1_ (6.0 μL, 10 μg/mL) and NMP-22 (6.0 μL, 10 ng/mL) were used to fabricate the immunosensors. As shown in Fig. 5(a), it could be found that the current response increased with increasing pH value from 5.8 to 7.0 to reach the maximum and decreased from 7.0 to 7.9. The reason is that the highly acidic or alkaline surroundings would damage the activity of immobilized protein^42^. Therefore, pH 7.0 PBS was selected for the test throughout this study.

Apart from the pH value of substrate solution, the concentration of rGO-NH was also an important parameter, which could affect not only the loading of captured antibody (Ab_1_) but also the electrochemical behaviors of the rGO-NH modified electrode. In detail, different concentrations of rGO-NH from 0.5 mg/mL to 2.0 mg/mL with the same amount of Ab_1_ (6.0 μL, 10 μg/mL) and NMP-22 (6.0 μL, 10 ng/mL) were employed to modify the electrodes. As seen in Fig. 5(b), with the increasing concentration of rGO-NH, the current response for detection of 10 ng/mL NMP-22 increased due to the enhanced loading of Ab_1_ for bound more antigens and then NH_2_-SAPO-34-Pd/Co-Ab_2_. However, when the concentration of the rGO-NH was more than 1.5 mg/mL, the current response decreased. Therefore, the optimal concentration of rGO-NH solution was 1.5 mg/mL.

**Figure 6:**
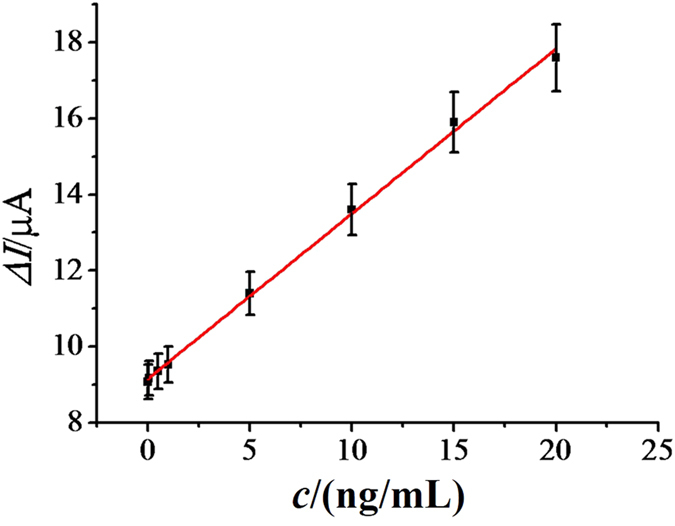
Calibration curve of the immunosensor toward different concentrations of NMP-22 (0.001, 0.05, 0.5, 1, 5, 10, 15, 20 ng/mL). Error bar = RSD (*n* = 5).

Under the optimum conditions, the immunosensors using NH_2_-SAPO-34-Pd/Co NPs as labels were prepared for the detection of different concentrations of NMP-22 in pH 7.0 PBS at −0.4 V. The relationship between the current response toward 5.0 mmol/L H_2_O_2_ and NMP-22 concentration was shown in Fig. 6. As can be seen, the current response increased linearly with the increasing concentration of the NMP-22 in the range from 0.001 to 20 ng/mL, with a detection limit of 0.33 pg/mL based on S/N = 3. The detection limit of this immunosensor is significantly lower than other methods^43^,^44^,^45^, as shown in Table 1. The calibration curve was linear with a correlation coefficient of R^2^ = 0.998 (Δ*I* = 0.434 *c* + 9.16).

To the further development of techniques, lower limit of detection is a major criterion of successful application. The low detection limit may be attributed to three factors: (1) The rGO-NH with large surface area could greatly increase the loading of Ab_1_ and promote electrons transfer because of its good conductivity; (2) SAPO-34 molecular sieves with large surface area could increase the loading of Pd/Co nanoparticles, which means more Ab_2_ could be loaded onto the label; (3) The good catalytic activity of NH_2_-SAPO-34-Pd/Co-Ab_2_ toward H_2_O_2_ could increase the sensitivity of the immunosensor. Hence, the proposed strategy could provide a stable immobilization and sensitized recognition platform for analytes such as micromolecules and possesses promising application in clinical sample.

### Reproducibility, selectivity and stability

To evaluate the reproducibility of the immunosensor, a series of five electrodes were prepared for the detection of NMP-22 (5 ng/mL). The results of the measurements were 11.7, 11.1, 11.9, 10.8 and 11.0 μA, respectively. The relative standard deviation (RSD) of the measurements for the five electrodes was 4.2%, suggesting the precision and reproducibility of the proposed immunosensor were good.

**Figure 7:**
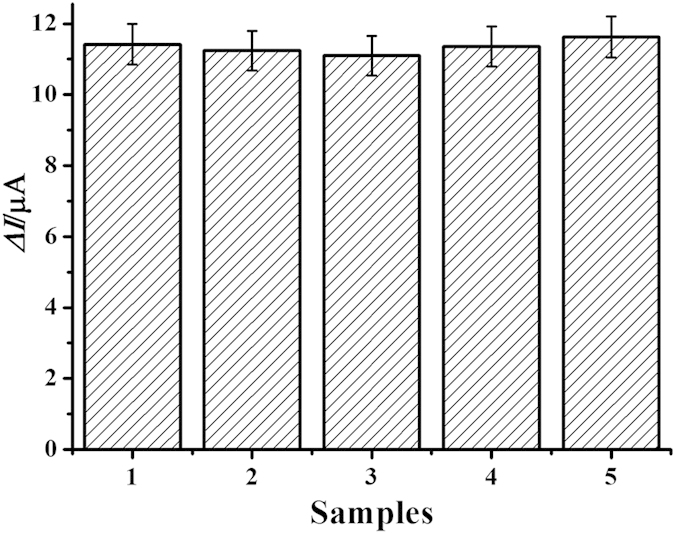
Amperometric response of the immunosensor to 5 ng/mL NMP-22 (1), 5 ng/mL NMP-22 + 500 ng/mL BSA (2), 5 ng/mL NMP-22 + 500 ng/mL vitamin C (3), 5 ng/mL NMP-22 + 500 ng/mL trioxypurine (4), 5 ng/mL NMP-22 + 500 ng/mL glucose (5). Error bar: RSD (*n* = 5).

The selectivity of the immunosensor was also investigated. Interferences study was performed by using bovine serum albumin (BSA), vitamin C, trioxypurine and glucose. NMP-22 (5 ng/mL) solutions containing 500 ng/mL of interfering substances were measured by the immunosensor. The results were shown in Fig. 7. The current variation due to the interfering substances was less than 5.0% of that without interferences, indicating that the selectivity of the immunosensor was acceptable.

Stability of the immunosensor is also a key factor in their application and development. The stability of the immunosensor for 5 ng/mL NMP-22 was examined by checking periodically its current response. When the immunosensor was not used, it was stored at 4 °C. After ten days, the current of the immunosensor retained about 97% of its initial value. The good long-term stability may be ascribed to the good stability of the NH_2_-SAPO-34-Pd/Co and rGO-NH.

### Real sample analysis

To evaluate the potential of this immunosensor for real sample analysis, the practical detection for two urine samples covered by the calibration curve is further conducted. As shown in Table 2, the RSD between 1.6% and 6.2% were obtained. The recovery was in the range from 99.5% to 101.2%. Therefore, the immunosensor could be used in the clinical analysis.

## Conclusion

The large surface area of rGO-NH could increase the amount of Ab_1_ immobilized on the electrode surface and the good conductivity of rGO-NH could promote the electrons transfer. The NH_2_-SAPO-34 supported Pd/Co nanoparticles showed high catalysis toward the reduction of H_2_O_2_, which improve the sensitivity of the immunosensor. The immunosensor has adequate sensitivity and precision, with wide linear range and low detection limit of 0.33 pg/mL. Due to the advantages of simplicity, high selectivity and good reproducibility, this new immunosensor may have the potential application in the detection of different cancer biomarkers.

## Additional Information

**How to cite this article**: Wu, D. *et al*. Sensitive Electrochemical Immunosensor for Detection of Nuclear Matrix Protein-22 based on NH_2_-SAPO-34 Supported Pd/Co Nanoparticles. *Sci. Rep*. **6**, 24551; doi: 10.1038/srep24551 (2016).

## Figures and Tables

**Table 1:** Comparisons of proposed method with other reports for NMP-22.

Methods	Linear range	Detection limit	Reference
Electrochemical immuno-biosensor	1.2–200 ng/mL	0.5 ng/mL	43
Electrochemical sensing	128–588 ng/mL	–	44
Label-free electrochemiluminescence immunosensor	0.05–2.0 ng/mL	10.0 pg/mL	45
Electrochemical immunosensor	0.001–20 ng/mL	0.33 pg/mL	This work

**Table 2:** Results for the determination of NMP-22 in the clinical urine sample by the immunosensor.

Urine Sample	Content ofNMP-22 (ng/mL)	Added(ng/mL)	Found(n = 5 ng/mL)	RSD(%, n = 5)	Recovery(%, n = 5)
1	2.99	5.00	7.79 8.11 8.05 7.87 7.95	1.6	99.5
2	0.73	3.00	4.12 3.79 3.50 3.63 3.84	6.2	101.2
